# Characterization of the absorption, metabolism, excretion, and mass balance of gefapixant in humans

**DOI:** 10.1002/prp2.924

**Published:** 2022-02-01

**Authors:** Jesse C. Nussbaum, Azher Hussain, Bennett Ma, K. Chris Min, Qing Chen, Charles Tomek, Marian Iwamoto, S. Aubrey Stoch

**Affiliations:** ^1^ Merck & Co., Inc. Kenilworth New Jersey USA; ^2^ Celerion Inc. Lincoln Nebraska USA

**Keywords:** AF‐219, antitussives, chronic cough, purinergic receptor, RO4926219

## Abstract

Gefapixant (MK‐7264) is a first‐in‐class, selective antagonist of the P2X3 purinergic receptor currently being investigated as a therapeutic agent for the treatment of refractory or unexplained chronic cough. In non‐clinical studies, gefapixant was eliminated primarily by renal excretion of the parent drug. The objective of this study was to assess the disposition of gefapixant in humans. The absorption, metabolism, and excretion profiles of gefapixant were assessed after oral administration of a single dose of [^14^C]gefapixant to six healthy adult males. Following a single‐oral [^14^C]gefapixant dose to healthy adult males, the mass balance was achieved, with 98.9% of the administered radioactivity recovered in urine and feces. Elimination of gefapixant occurred primarily via renal excretion of the intact drug (64%); metabolism was a minor pathway of elimination of gefapixant (12% and 2% recovered in urine and feces, respectively). Single‐dose administration of [^14^C]gefapixant 50 mg was generally well tolerated in healthy adult males. The fraction of the anticipated therapeutic oral dose of gefapixant absorbed is estimated to be at least 78%. Gefapixant is expected to be the major circulating drug‐related material in plasma, and the majority of the dosed drug will be excreted unchanged in urine.

**Abbreviations**
%Dose: Ae/administered dose × 100
ACV: arithmetic percent coefficient of variation
AE: adverse event
Ae: the total concentration equivalents × weight in the given collection interval
AM: arithmetic mean
AUC: area under the concentration vs. time curve
AUC_0–24_
: AUC from pre‐dose to 24 h post‐dose
AUC_0–_ **_∞_**
: AUC from pre‐dose extrapolated to infinity
AUC_0–last_
: AUC from pre‐dose to the time of the last quantifiable sample
CL_r_
: renal clearance
C_max_
: maximum plasma concentration
Cum%Dose: cumulative sum of the %Dose
Cum_Ae_
: cumulative sum of Ae
CYP: cytochrome P450
DDI: drug‐drug interaction
GCV: geometric percent coefficient of variation
GM: Geometric mean
GMRs: geometric mean ratios
LC‐HRMS: liquid chromatography‐high resolution mass spectrometry
LLOQ: lower limit of quantitation
LSC: liquid scintillation counting
M1: glucuronic acid conjugate
M11: oxidative metabolite
M13: mono‐hydroxylated metabolite
M5: glucuronic acid conjugate of the parent
M6: *O*‐demethylated metabolite
M8: dehydrogenated metabolite
MATE: multidrug and toxin extrusion transporters
PK: pharmacokinetics
t_1/2_
: terminal half‐life
T_last_
: last collection time at which drug is observed
T_max_
: time to reach C_max_
UGT: uridine 5’‐diphosphoglucuronic acid glucuronosyl transferase

**SIGNIFICANCE STATEMENT**

[^14^C]‐labeled gefapixant elimination occurs primarily via renal excretion of the intact drug, and metabolism is a minor pathway of elimination. By combining the fractions eliminated by different routes, the fraction of an oral dose of gefapixant that is absorbed is estimated to be at least 78%. The findings in this study enable a targeted approach to further characterization of gefapixant clinical pharmacology, including an estimation of the absorbed fraction without an intravenous dose.

## 1 INTRODUCTION

The global prevalence of chronic cough (a cough persisting longer than 8 weeks) has been estimated to be approximately 10%.^1^ Although the cough reflex is typically protective, chronic cough can negatively impact both the physical and emotional well‐being of affected individuals.^2^ Gefapixant (MK‐7264) is a first‐in‐class, selective antagonist of the P2X3 purinergic receptor that is currently under investigation as a therapeutic agent for the treatment of refractory or unexplained chronic cough.^3^ The P2X3 receptor is an adenosine triphosphate (ATP)‐gated ion channel that can be found on sensory nerves.^4^ ATP released by damaged, stressed, or inflamed tissues in airways stimulates C fiber sensory nerves through P2X3, initiating a cough reflex.^5^ Blockade of extracellular ATP signaling through P2X3 receptors reduces sensory‐nerve activation and cough in pre‐clinical models,^6^, ^7^, ^8^ and data from clinical trials suggest gefapixant can have a similar effect in patients.^9^, ^10^, ^11^

In non‐clinical studies in animals, gefapixant was eliminated primarily by renal excretion of the parent drug. Here, we report the results of a Phase 1 clinical trial designed to assess the disposition of gefapixant in humans. In this study, the absorption, metabolism, and excretion profiles of gefapixant were assessed after oral administration of a single dose of [^14^C]gefapixant to healthy adult males.

## 2 MATERIALS AND METHODS

The study reported here was approved by Chesapeake Research Review, Inc., MA, USA and conducted at Celerion Inc., Lincoln, NE, USA. The study was conducted in accordance with Good Clinical Practices guidelines and the ethical principles set forth by the Declaration of Helsinki. All participants gave written informed consent.

### 2.1 Materials

[^14^C]gefapixant was synthesized by the Merck Labelled Compound Synthesis group (Rahway, NJ, USA), with ^14^C incorporated in the diaminopyrimidine ring. An oral suspension of [^14^C]gefapixant powder was formulated in Ora‐Blend^®^ SF (Perrigo, Allegan, Michigan, USA), at a target concentration of 10 mg/mL with a specific activity of approximately 4 μCi/mg.

### 2.2 Participants

Eligible study participants were male, 19 to 45 years of age, with a body mass index (BMI) ≥18.5 and ≤32.0 kg/m², and in good health based on medical history, physical examination, laboratory safety tests, vital sign measurements, and electrocardiogram (ECG) assessments. Individuals with a history or presence of hypersensitivity or idiosyncratic reaction to the study drug or related compounds were excluded from the trial.

### 2.3 Study design

This was a single‐center, single‐group assignment, open‐label, study. Following an overnight fast, participants received a single oral dose of 50 mg [^14^C]gefapixant, equivalent to approximately 200 μCi, a dose selected to deliver sufficient radioactivity for analysis and to be within the linear range of the gefapixant dose‐exposure relationship. Blood, urine, and fecal samples were collected for radioactivity and pharmacokinetic assessments. Safety was monitored throughout, including collection and evaluation of adverse events (AEs), vital signs, ECGs, and clinical laboratory evaluations (hematology, chemistry, and urinalysis). Participants were discharged within 7 days post‐dose, a timepoint at which ≥90% of the administered radioactivity had been recovered in urine and feces, and ≤1% of the administered radioactivity was found in each of 2 samples from consecutive 24‐h urine and fecal collections.

### 2.4 Sample collection and processing

Gefapixant concentrations were assessed in plasma and urine samples. Blood samples were collected pre‐dose and 0.5, 1, 1.5, 2, 3, 4, 6, 8, 12, 16, 24, 48, 72, 96, and 120 h post‐dose. Urine samples were collected pre‐dose and during the intervals of 0 to 4, 4 to 8, 8 to 12, 12 to 24 h, and then at 24‐h intervals until 120 h post‐dose. Fecal samples were collected pre‐dose (within 48 h of drug administration) and throughout the duration of domiciling. Fecal samples were homogenized and pooled by the participant over 24‐h intervals. All samples were kept on ice until frozen at −20°C then thawed for homogenization (fecal samples) or final processing.

### 2.5 Gefapixant quantification

Blood and urine samples were processed and assayed as previously described for gefapixant quantification.^12^ Plasma and urine gefapixant concentrations were determined by inVentiv Health Clinique (Quebec, Quebec, Canada) using a validated high‐performance liquid chromatographic tandem mass spectrometric method, with a lower limit of quantitation (LLOQ) of 10.00 ng/mL and analytical range of 10.00 to 10000.00 ng/mL for plasma and an LLOQ of 1.00 μg/mL and analytical range of 1.00 to 1000.00 μg/mL for urine.^12^

### 2.6 Radioactivity measurements

Total radioactivity concentration equivalents in plasma, urine, and feces were determined by Celerion (Lincoln, Nebraska, USA) using liquid scintillation counting (LSC). Radioactivity in urine and plasma was analyzed by direct counting of triplicate sample aliquots in vials containing liquid scintillation cocktail (Ultima Gold XR, PerkinElmer, Boston, MA, USA. Product No. 6013119). The aliquot mass for plasma was 0.25 g. The aliquot mass for urine was 1 g. The aliquot mass for fecal samples was 0.5 g. Fecal homogenate aliquots were dried and oxidized before assaying for [^14^C] content. The LLOQ for total radioactivity in plasma, urine, and feces were 19.2 ng eq/g, 4.99 ng eq/g, and 11.5 ng eq/g, respectively, LLOQ = {[(average background dpm × 2.5) – average background dpm]/grams average aliquot weight for all aliquots for all participants}/specific activity (dpm/gram). Further details of radioactivity analysis can be found in Supplemental Text.

### 2.7 Metabolite profiling

Plasma samples from all participants were pooled together according to the “Hamilton” time proportional pooling algorithm from 0.5–24 h post‐dose (representing approximately 85% of the entire Area Under the Concentration vs. time curve [AUC]).^13^ In addition, plasma samples from all participants at 2 h post‐dose were pooled together by equal volume into a single 2‐h sample. Urine samples from all participants (0–48 h post‐dose) were pooled together proportionally based on volumes. Selected fecal samples from each participant (included samples had >2% of the dose recovered in feces from each participant 0–144 h post‐dose) were pooled together proportionally based on the weight. Approximately 2 to 3 mL of plasma pools, 100 mL of urine pool, and 30 g of fecal homogenate pool were frozen at −20°C until shipment to Merck & Co. Inc. (Kenilworth, New Jersey, USA) for metabolite profiling. Metabolite profiling was performed using liquid chromatography‐high resolution mass spectrometry (LC‐HRMS) and radiometric detection. Metabolites present in trace amounts were detected by HRMS only. Further details on the extraction and metabolite profiling methods can be found in Supplemental Text. The overall extraction recovery for all samples was >90%.

### 2.8 Pharmacokinetic analysis

Any gefapixant or radioactivity concentrations from any source that was below the limits of quantitation were replaced with a value of 0 in all analyses.

#### 2.8.1 Gefapixant pharmacokinetics and radioactivity

Plasma gefapixant concentrations, total radioactivity concentration equivalents, and actual sampling times relative to [^14^C]gefapixant dosing time were used to determine the plasma pharmacokinetics (PK) of gefapixant and total radioactivity. All derived plasma PK parameter values were calculated using the software Phoenix^®^ WinNonlin^®^ (Version 6.3). AUCs were calculated using the linear trapezoidal method for ascending concentrations and the log trapezoidal method for descending concentrations (linear up, log down) calculation method. PK parameters assessed were AUC_0–24_ (AUC from pre‐dose to 24 h post‐dose, the last common time point at which gefapixant and total radioactivity were quantifiable in all participants), AUC_0–last_ (AUC from pre‐dose to the time of the last quantifiable sample), AUC_0–_ **_∞_** (AUC from pre‐dose extrapolated to infinity), C_max_ (maximum plasma concentration), T_max_ (time to reach C_max_), T_last_ (last collection time at which drug is observed), and the apparent terminal half‐life (t_1/2_). The ratios of plasma gefapixant to plasma total radioactivity for AUC_0–24_ and C_max_ were calculated.

The total gefapixant concentrations in urine, and the urine sample weights from individual collection intervals, were used to calculate the urinary gefapixant Ae (the total gefapixant concentration equivalents × urine weight in the given collection interval). Renal clearance (CL_r_) was calculated as the quotient of Ae_0–24_ of gefapixant in urine and AUC_0–24_ of plasma gefapixant (Ae_0–24_/AUC_0–24_) since 24 h post‐dose was the last common time point at which gefapixant in urine and plasma were quantifiable in all subjects.

Total radioactivity concentration equivalents in urine and feces, and the urine sample and fecal homogenate weights from individual collection intervals, were used to calculate the urine and fecal radioactivity Ae, Cum_Ae_ (the cumulative sum of Ae), %Dose (Ae/administered dose × 100) and Cum%Dose (cumulative sum of the %Dose) of radioactivity in urine and feces.

#### 2.8.2 Analyses for metabolites

The percent of dose represented by each of the metabolites was calculated using the radioactivity concentration‐equivalent data combined with the metabolite profiling data. The percentage of each identified metabolite to total radioactivity in the plasma was estimated based on plasma metabolite profiling data.

### 2.9 Statistical analyses

To estimate the proportion of total radioactivity accounted for by gefapixant in plasma, a linear mixed‐effects model was used with the analyte (gefapixant or total radioactivity) as a fixed‐effect and trial participant as a random‐effect. The individual values of AUC_0–24_, AUC_0–_ **_∞_**, and C_max_ were ln‐transformed and model‐based geometric means and 95% confidence intervals (CIs) for gefapixant and total radioactivity were calculated. AUC_0–24_ and C_max_ geometric mean ratios (GMRs, gefapixant/total radioactivity) were calculated, along with corresponding 95% CIs. GMRs and corresponding 95% CIs for AUC_0–_ **_∞_** were calculated post hoc.

Non‐model‐based descriptive statistics were used to characterize the sample size (*N*, the number of participants with non‐missing data), arithmetic mean (AM), standard deviation (SD), arithmetic percent coefficient of variation (ACV) (calculated as 100 × SD/AM), median (Med), minimum (Min), maximum (Max), geometric mean (GM), and geometric percent CV (GCV) (calculated as 100 × sqrt(exp(s2) ‐ 1), where s2 is the observed variance on the ln‐scale). GM and GCV were only calculated if all concentrations were above the LLOQ. When *N* was ≤2, SD was not calculated.

Non‐model‐based 95% CIs of the AM were calculated for Cum_Ae_ and Cum%Dose for the recovery of total radioactivity in urine, feces, and overall. AM (with SD) and individual plasma total radioactivity and gefapixant concentration‐time plots were generated. The AM concentration for a given time point was only presented when ≥50% of the participants had quantifiable concentration values and the resultant AM was >LLOQ. If <50% of participants had quantifiable concentration values for a given time point at the beginning of the profile, the AM was set to 0. If <50% of participants had quantifiable concentration values for a given time point at the end of the profile, the AM was set to missing.

### 2.10 Safety

Safety and tolerability were evaluated by clinical assessment of AEs, vital signs, 12‐lead ECGs, and laboratory safety tests (hematology, chemistry, and urinalysis) throughout the study.

## 3 RESULTS

### 3.1 Participant demographics and disposition

The trial was initiated July 14, 2017 and completed August 9, 2017. Six healthy adult males were enrolled in the study and five completed the study per protocol. One participant was considered lost to follow‐up and discontinued from the study after completing all study procedures with the exception of the follow‐up call. Data from this participant were included in all analyses. The mean (range) age and BMI of the participants were 27.8 years (22 to 43) and 25.3 kg/m^2^ (21.0 to 30.2), respectively. None of the participants were of Hispanic or Latino ethnicity, and the distribution of race was 33.3% Black or African American and 66.7% white.

### 3.2 Pharmacokinetics of gefapixant

**FIGURE 1:**
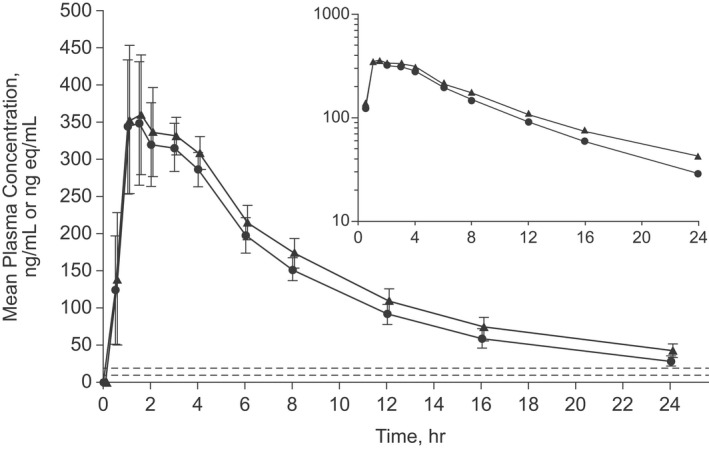
Arithmetic mean (± standard deviation) plasma concentration‐time profiles of gefapixant (●) and total radioactivity (▲) in plasma following the administration of a single oral dose of [^14^C]gefapixant 50 mg (approximately 200 μCi) to healthy adult males (*N *= 6). The inset shows the data plotted on a semi‐logarithmic scale. The arithmetic mean concentrations after 24 h post‐dose are not presented for either gefapixant or radioactivity because concentrations of both in all participants were less than the lower limit of quantitation (dashed lines, 10.0 ng/mL for gefapixant and 19.2 ng eq/mL for total radioactivity) after this time

The plasma concentration‐time profiles of gefapixant and of total radioactivity in plasma following a single oral dose of [^14^C]gefapixant 50 mg (approximately 200 μCi) were similar (Figure 1) and corresponding PK parameter values are summarized in Table 1. The geometric least‐squares mean AUC_0–24_, AUC_0–∞_, and C_max_ values for gefapixant/total radioactivity were 0.88, 0.84, and 0.97, respectively. The median T_max_ values for both plasma gefapixant and total radioactivity were 1.5 h, and the t_1/2_ values were similar (Table 1).

**TABLE 1:** Pharmacokinetic parameter values of gefapixant and recovery of radioactivity following a single oral dose of [^14^C]gefapixant 50 mg (approximately 200 μCi) administered to healthy male participants (*N* = 6)

Parameters	Gefapixant	Total radioactivity	Gefapixant/ Total radioactivity^a^
AUC_0–24_, ng·hr/mL or ng eq·hr/mL	3030 (2700, 3390)	3430 (3060, 3840)	0.88 (0.85, 0.92)
AUC_0–∞_, ng·hr/mL or ng eq·hr/mL	3310 (2910, 3780)	3930 (3450, 4480)	0.84 (0.80, 0.88)
C_max_, ng/mL or ng eq/mL	369 (314, 434)	381 (325, 448)	0.97 (0.92, 1.02)
*T*_max_ ^b^, hr	1.5 (1.0, 4.0)	1.5 (1.0, 4.0)	—
*t*_½_ ^c^, hr	7.1 (7.2)	8.3 (17.6)	—

Radioactive dose recovered			
	Urine	Feces	Total
Fraction of radioactivity recovered^d^, %	76.4 (72.5, 80.2)	22.6 (18.4, 26.8)	98.9 (98.0, 99.9)

Notes

Unless noted otherwise, values are geometric least‐squares mean (95% Confidence Interval [CI]) from linear mixed‐effects model performed on natural log‐transformed values.

AUC = the area under the concentration‐time curve; AUC_0–24_ = AUC from pre‐dose to 24 h post‐dose (the last common time point at which gefapixant and total radioactivity were quantifiable in all participants); AUC_0–∞_ = AUC from pre‐dose extrapolated to infinity; C_max_ = maximum observed plasma concentration; T_max_ = time to reach C_max_; T_last_ = time of the last quantifiable sample; t_1/2_ = apparent terminal half‐life

^a^ Ratio of geometric least‐squares means of gefapixant with respect to total radioactivity (95%CI)

^b^ Median (min, max)

^c^ Geometric mean (geometric coefficient of variation [GCV]); GCV is calculated in the natural log scale with the equation: 100*sqrt[exp(s2)‐1] where s2 is the observed variance on the natural log scale.

^d^ Arithmetic mean (95% CI)

### 3.3 Excretion of gefapixant

**FIGURE 2:**
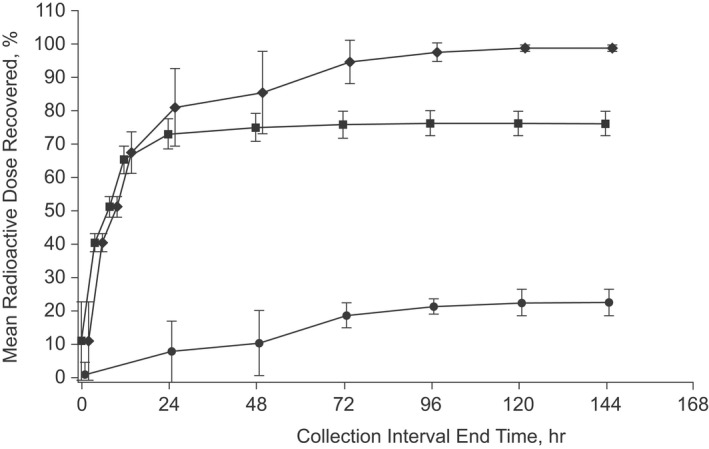
Cumulative arithmetic mean percent (±standard deviation) of urine (■), fecal (●), and total (⧫) recovery of drug‐derived radioactivity following administration of a single oral dose of [^14^C]gefapixant 50 mg (approximately 200 μCi) to healthy adult males (*N* = 6)

Following oral administration of [^14^C]gefapixant 50 mg (approximately 200 μCi), urine and feces were collected through 5 days (120 h) post‐dose in all but one participant, who had samples collected up to 7 days (168 h) post‐dose. Mass balance was achieved, with 99% of the administered radioactivity recovered in urine and feces over 1 week. Of radioactivity recovered, approximately 76% was in urine and approximately 23% was in feces (Table 1, Figure 2). The Cum_Ae_ was 28.6 mg which accounted for approximately 57.3% of the Cum%Dose; GM CL_r_ of gefapixant was approximately 8.68 L/hr.

### 3.4 Metabolite profiles

**FIGURE 3:**
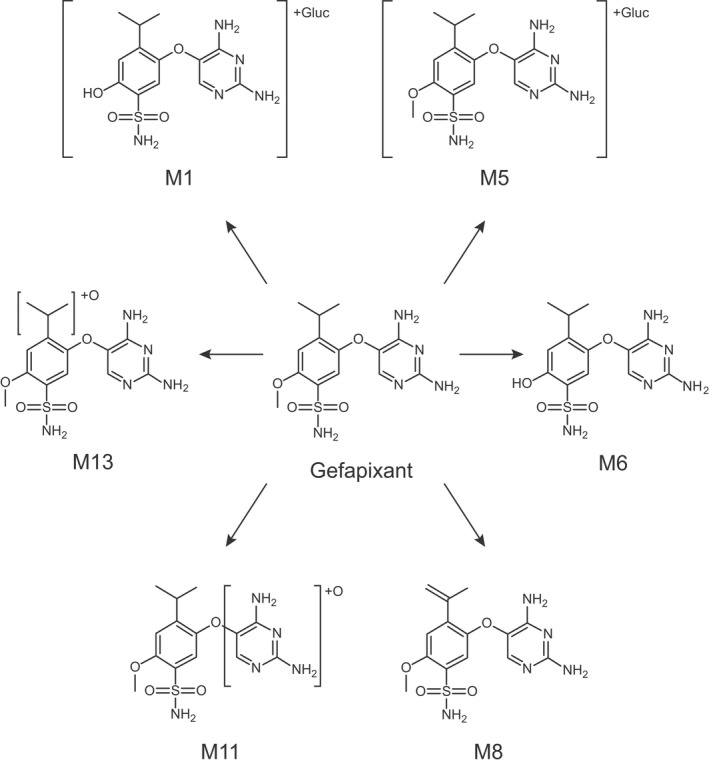
Proposed Structures of Human Metabolites of gefapixant

The results of metabolite profiling indicated that parent molecule (gefapixant) accounted for 64% and 20% of the dose in urine and feces, respectively. In urine, small amounts of a direct glucuronic acid conjugate of the parent (M5), an oxidative metabolite (M11), and a mono‐hydroxylated metabolite (M13) were detected, with each metabolite representing <5% of the dose (Figure 3; Table 2). Other minor metabolites detected in urine included a dehydrogenated metabolite (M8) and a glucuronic acid conjugate (M1) of the *O*‐demethylated metabolite (M6) (Figure 3). The metabolites detected in pooled feces were M5, M6, M11, and M13, and each metabolite represented <1% of the dose (Table 2). Overall, metabolites detected in urine and feces collectively accounted for <15% of the dose (Table 2). In plasma, M1, M5, M8, M11, and M13 were detected as minor metabolites (Figure 3). Each circulating metabolite accounted for less than 10% of the total radioactivity detected (Table 2).

**TABLE 2:** Summary of radioactivity percentage of gefapixant and metabolites detected in healthy adult males (*N *= 6) following a single oral dose administration of [^14^C]gefapixant 50 mg (~200 μCi)

Molecule	Plasma (2 h)	Plasma (0.5 to 24 h)	Urine (0 to 48 h)	Feces (0 to 168 h)
Gefapixant, parent	93	87	84 (64)	90 (20)
M1 parent‐CH_2_+Gluc	n.d.	1.0	2.1 (1.6)	n.d.
M5 parent+Gluc	2.0	6.3	5.5 (4.2)	3.1 (0.7)
M6 parent‐CH_2_	n.d.	n.d.	n.d.	2.3 (0.5)
M8 parent‐H_2_	trace	trace	0.6 (0.5)	n.d.
M11 parent +O	1.5	n.d.	2.3 (1.8)	1.4 (0.3)
M13 parent +O	3.4	5.8	5.2 (4.0)	3.4 (0.8)

Note

Values represent the percentage of radioactivity in the radiochromatogram associated with parent compound and metabolites. Numbers in parentheses represent the percentage of the dose. Metabolites that were detected in trace amounts were detected by high‐resolution mass spectrometry only.

Abbreviations: n.d., not detected.

### 3.5 Safety

Single‐dose administration of [^14^C]gefapixant 50 mg (approximately 200 μCi) was generally well tolerated in healthy adult males. Two of the six participants reported a total of three treatment‐emergent AEs, all of which were assessed by the investigator as related to the study drug. Mild headache was reported by two participants and mild oral hypoesthesia was also reported by one of these participants. There were no deaths, serious AEs, or discontinuation due to AEs during the study. No clinically meaningful relationships were observed for changes in clinical laboratory values, vital signs, or ECGs.

## 4 DISCUSSION

Gefapixant is a P2X3 receptor antagonist being developed as a treatment for refractory or unexplained chronic cough. Maximal suppression of chronic cough by gefapixant has been observed at doses of approximately 50 mg BID^11^ with the 45‐mg dose BID projected as the therapeutic dose.

The primary purpose of this study was to investigate the absorption, metabolism, excretion, and mass balance of gefapixant after oral administration. The total recovery of radioactivity over the period of 168 h was nearly complete, accounting for approximately 99% of the dose. Approximately 20% of the dose was excreted unchanged in feces, and while it is not clear if this represents unabsorbed drug or biliary excretion, based on the dose recovered in urine (76%) and the dose recovered as metabolites in feces (2%), the fraction absorbed for gefapixant is estimated to be at least 78%.

In urine, 64% of the dose was recovered as unchanged gefapixant, indicating gefapixant is mainly eliminated via renal excretion of the unchanged drug. In vitro studies showed that gefapixant is a substrate of multidrug and toxin extrusion transporters 1 and 2 (MATE1 and MATE2K).^12^ In a drug‐drug interaction (DDI) study conducted with co‐administration of gefapixant and pyrimethamine, a competitive inhibitor of MATE1 and MATE2K, a 24% increase in total gefapixant plasma exposure was observed with the concomitant dosing of pyrimethamine. These results indicated that MATE1 and/or MATE2K plays a role in the renal clearance of gefapixant in addition to contributions from passive filtration and/or other efflux transporters; however, there is low potential for gefapixant to be a victim of meaningful DDIs with inhibitors of MATE1 and/or MATE2K.

Approximately 14% of the dose was excreted as metabolites (12% in urine and 2% in feces), indicating that metabolism is a minor pathway for the elimination of gefapixant. The metabolites detected in urine and feces indicated gefapixant underwent oxidation as well as direct glucuronidation. Given that metabolism is a minor clearance mechanism, the potential for clinically relevant DDI for gefapixant with co‐administration of inhibitors or inducers of cytochrome P450 (CYP) or uridine 5’‐diphosphoglucuronic acid glucuronosyltransferase (UGT) enzymes is low.

Gefapixant was the major drug‐related component in plasma following the administration of a single oral dose of [^14^C]gefapixant 50 mg (approximately 200 μCi), as determined both by the ratio of gefapixant AUC to total radioactivity AUC (88% through 24 h, Table 1) and by the analysis of metabolite profiling in plasma (87% of parent in samples pooled from 0.5 to 24 h, Table 2). This observation further supports that metabolism plays a minor role in the elimination of gefapixant, and the exposure of each of the circulating metabolites was less than 10% of the total drug‐related exposure. All metabolites detected in human excreta and plasma have been observed previously in either rats and/or dogs.

The design of the study carries some limitations. Only single‐dose administration at a single dose level was assessed; however, the PK properties of gefapixant are not time dependent and exposures are dose proportional in this range; therefore, single‐dose PK can be used to estimate steady‐state PK. The observed safety, while limited by the open‐label design of this study, is consistent with previous studies of gefapixant indicating that it is generally well tolerated.

In conclusion, following a single oral [^14^C]gefapixant dose to healthy adult males, the mass balance was achieved, with 98.9% of the administered radioactivity recovered in urine and feces. In healthy male adults, elimination of gefapixant occurs primarily via renal excretion of the intact drug (64%); metabolism is a minor pathway of elimination of gefapixant (12% and 2% recovered in urine and feces, respectively). Based on these values, the fraction of drug absorbed is estimated to be at least 78%. Gefapixant is the major circulating drug‐related material in plasma. Single‐dose administration of [^14^C]gefapixant 50 mg was generally well tolerated in healthy adult males.

## CONFLICT OF INTEREST

JCN, AH, BM, KCM, QC, MI, and SAS are current or former employees of Merck Sharp & Dohme Corp., a subsidiary of Merck & Co., Inc., Kenilworth, NJ, USA and may own stock/stock options in Merck & Co., Inc., Kenilworth, NJ, USA. CT has no disclosures. KCM is currently employed by Neurologic Insight LLC, New York, NY, USA. QC is currently employed by Vertex Pharmaceuticals, Boston, MA, USA.

## AUTHOR CONTRIBUTIONS

All authors are responsible for the work described in this paper. Conceived, designed, and/or planned the study: Hussain, Min, Chen, and Stoch. Acquired the data: Chen and Tomek. Analyzed the data: Nussbaum, Chen, and Stoch. Interpreted the results: Nussbaum, Hussain, Ma, Chen, Tomek, Iwamoto, and Stoch. Drafted the manuscript: Nussbaum and Ma. All authors critically reviewed and/or revised the manuscript for important intellectual content, provided final approval of the version to be published, and agree to be accountable for all aspects of the work in ensuring that questions related to the accuracy or integrity of any part of the work are appropriately investigated and resolved.

## Supporting information

Supplementary Material

## Data Availability

The data sharing policy, including restrictions, of Merck Sharp & Dohme Corp., a subsidiary of Merck & Co., Inc., Kenilworth, NJ, USA is available at http://engagezone.msd.com/ds_documentation.php. Requests for access to the clinical study data can be submitted through the Engage Zone site or via email to dataaccess@merck.com.

## References

[1] Song W‐J , Chang Y‐S , Faruqi S , et al. The global epidemiology of chronic cough in adults: a systematic review and meta‐analysis. Eur Respir J. 2015;45(5):1479‐1481. 25657027 10.1183/09031936.00218714

[2] Chung KF , McGarvey L , Mazzone SB . Chronic cough as a neuropathic disorder. Lancet Respir Med. 2013;1(5):414‐422. 24429206 10.1016/S2213-2600(13)70043-2

[3] Muccino D , Green S . Update on the clinical development of gefapixant, a P2X3 receptor antagonist for the treatment of refractory chronic cough. Pulm Pharmacol Ther. 2019;56:75‐78. 30880151 10.1016/j.pupt.2019.03.006

[4] Hermes SM , Andresen MC , Aicher SA . Localization of TRPV1 and P2X3 in unmyelinated and myelinated vagal afferents in the rat. J Chem Neuroanat. 2016;72:1‐7. 26706222 10.1016/j.jchemneu.2015.12.003 PMC4764453

[5] Dicpinigaitis PV , McGarvey LP , Canning BJ . P2X3‐receptor antagonists as potential antitussives: summary of current clinical trials in chronic cough. Lung. 2020;198(4):609‐616. 32661659 10.1007/s00408-020-00377-8

[6] Weigand LA , Ford AP , Undem BJ . A role for ATP in bronchoconstriction‐induced activation of guinea pig vagal intrapulmonary C‐fibres. J Physiol. 2012;590(16):4109‐4120. 22687618 10.1113/jphysiol.2012.233460 PMC3476651

[7] Ford AP . In pursuit of P2X3 antagonists: novel therapeutics for chronic pain and afferent sensitization. Purinergic Signal. 2012;8(Suppl 1):3‐26. 22095157 10.1007/s11302-011-9271-6 PMC3265711

[8] Kwong K , Kollarik M , Nassenstein C , Ru F , Undem BJ . P2X2 receptors differentiate placodal vs. neural crest C‐fiber phenotypes innervating guinea pig lungs and esophagus. Am J Physiol Lung Cell Mol Physiol. 2008;295(5):L858‐L865. 18689601 10.1152/ajplung.90360.2008 PMC2584877

[9] Abdulqawi R , Dockry R , Holt K , et al. P2X3 receptor antagonist (AF‐219) in refractory chronic cough: a randomised, double‐blind, placebo‐controlled phase 2 study. Lancet. 2015;385(9974):1198‐1205. 25467586 10.1016/S0140-6736(14)61255-1

[10] Smith JA , Kitt MM , Butera P , et al. Gefapixant in two randomised dose‐escalation studies in chronic cough. Eur Respir J. 2020;55(3):1901615. 31949115 10.1183/13993003.01615-2019

[11] Smith JA , Kitt MM , Morice AH , et al. Gefapixant, a P2X3 receptor antagonist, for the treatment of refractory or unexplained chronic cough: a randomised, double‐blind, controlled, parallel‐group, phase 2b trial. Lancet Respir Med. 2020;8(8):775‐785. 32109425 10.1016/S2213-2600(19)30471-0

[12] Nussbaum JC , Hussain A , Ma B , et al. Assessment of the effect of pyrimethamine, a potent inhibitor of multidrug and toxin extrusion protein 1/2K, on the pharmacokinetics of gefapixant (MK‐7264), a P2X3 receptor antagonist. Clin Pharmacol Drug Dev. 2022;11(1):123‐128. 34145987 10.1002/cpdd.988

[13] Hamilton RA , Garnett WR , Kline BJ . Determination of mean valproic acid serum level by assay of a single pooled sample. Clin Pharmacol Ther. 1981;29(3):408‐413. 6781809 10.1038/clpt.1981.56

