# Supplementary material for: Characterization of the absorption, metabolism, excretion, and mass balance of gefapixant in humans
Source: Pharmacol Res Perspect. 2022 Feb 1;10(1):e00924. doi: 10.1002/prp2.924 (PMC8929362; doi:10.1002/prp2.924)
Supplement: Supplementary file 1 — Supplementary Material [file PRP2-10-e00924-s001.docx]

**SUPPLEMENTARY MATERIAL**

**Characterization of the Absorption, Metabolism, Excretion, and Mass Balance of Gefapixant in Humans**

Jesse C. Nussbaum*, Azher Hussain, Bennett Ma, K. Chris Min, Qing Chen, Charles Tomek, Marian Iwamoto, S. Aubrey Stoch

Table of Contents:

*Supplemental: Radioactivity analysis page 2*

*Supplemental: Processing of Samples for Metabolite Profiling page 3*

*Supplemental: Metabolite Bioanalysis page 5*

*Supplemental: Radioactivity analysis*

All samples were analyzed for total radioactivity content by LSC using counters manufactured by PerkinElmer (Waltham, MA). Urine (1 g) and plasma (0.25 g) samples were analyzed by direct counting of weighed triplicate sample aliquots in vials containing 10 mL of Ultima Gold liquid scintillation cocktail (PerkinElmer). Fecal samples were homogenized at an approximately 1:4 dilution in 20% methanol in water. Samples of homogenized fecal material (0.5 g) were dried and oxidized with an oxidizer (PerkinElmer). A volume of 10 mL of Carbosorb® was used to sequester ^14^CO_2_ during oxidation. A volume of 10 mL of Permafluor®E (PerkinElmer) was used as the liquid scintillation cocktail. In all cases an acquisition time of 5 minutes was used. The acceptance criterion for blank samples was ≤100 dpm, and the precision of spiked quality control samples were within 15% of theoretical radioactivity. The acceptance criterion for replicate samples was that each replicate must be within 15% of the mean value of the replicates, with the exception of blanks or clinical samples for which the average of the replicates was less than 200 dpm.

*Supplemental: Processing of Samples for Metabolite Profiling*

Plasma samples were pooled according to the “Hamilton” time proportional pooling algorithm from 0.5-24 h (representing ~85% of AUC) and across participants so that AUC was equal to C_pool_ x ΔT (Hamilton et al. (1981) *Clin Pharmacol Ther* **29**:408-41); plasma samples from 2 hours post-dose from all participants were pooled by equal volume to create a single pooled sample. Pooled plasma samples (1 mL each) were first precipitated with 3 mL of acetonitrile:methanol (50:50, v/v), followed by vigorous agitation with a vortex and sonication for 10 min each, and centrifugation at 3000 g for 15 min at 4°C. The pellets were re-suspended in water (1 mL), and re-precipitated by 2 mL of acetonitrile:methanol (50:50, v/v). The supernatants from the two extractions were combined and dried down under flow of N2 at 35°C. Dried extract was reconstituted with ~150 μL of water/acetonitrile/methanol (70:15:15, v/v/v), agitated with a vortex, centrifuged at 13000 rpm for 5 min and supernatant was transferred to an HPLC vial for analysis.

Urine samples from each participant, representing >95% of total radioactivity recovered from each participant in urine (0-48 hours), were pooled proportionally (based on volume). Pooled urine sample (2 mL) was precipitated with two volumes of acetonitrile/methanol (50:50, v/v), followed by vigorous agitation with a vortex and sonication for 10 min each, and then centrifugation for 15 min at 3000 g at 4°C. The pellets were re-suspended in water (0.5 mL) and re-precipitated by the same method. The combined supernatants from the two precipitations were dried under a flow of N_2_ at 35°C. Sample was dissolved with approximately 380 μL of water/acetonitrile/methanol (70:15:15: v/v/v), followed by vigorous agitation with a vortex and sonication, and then centrifugation at 14000 rpm (Eppendorf centrifuge, model 5417C) for 5 min. The supernatant was transferred to an HPLC vial for analysis.

Fecal samples (representing >95% of total radioactivity recovered in feces) were pooled proportionally based on the weight from the following selected samples of 6 participants (samples with greater than 2% of the dose recovered in feces): Subject 0001, 72-96 hours; Subject 0002, 72-144 hours; Subject 0003, 24-48 hours; Subject 0004, 0-72 hours; Subject 0005, 48-96 hours; Subject 0006, 24-48 hours and 72-120 hours. The pooled fecal sample (2 mL) was extracted as described above for the pooled urine sample and dissolved in 550 μL of water/acetonitrile/methanol for HPLC analysis.

*Supplemental: Metabolite Bioanalysis*

Chromatographic separation of gefapixant and its metabolites was conducted using an Acquity UPLC system (Waters, Milford, MA) with an Agilent Zorbax SB-C8 column (4.6 mm x 250 mm, 5 µm) (Agilent Technologies, INC. Santa Clara, CA). The mobile phase A consisted of 0.1% formic acid in water and the mobile phase B consisted of 0.1% formic acid in acetonitrile. The flow rate was held constant at 1 mL/min. The gradient was 5% B at 0 min - 1 min, 20% B at 30 min, 100% B at 35 – 40 min, 5% B at 40.1 – 45 min.

A portion (3/4) of the LC effluent from the UPLC was diverted to fraction collection (Gilson FC204 fraction collector) into Wallac 96-well Scintiplate microplates at 0.2 min intervals per fraction. Another portion (1/4) was diverted to the mass spectrometer. Samples in the microplates were dried under flow of N_2_ at 35°C, then radioactivity was measured using a Wallac Microbeta scintillation counter (10 min per well). Reconstruction of radiochromatograms was completed using Laura software (LabLogic Systems Ltd., Sheffield, UK, version 4.1.1.26 SP3).

The MS analysis was conducted using a Xevo Q-Tof G2 mass spectrometer (Waters, Milford, MA) equipped with an electrospray ionization source and operated in the positive ionization mode. High resolution mass spectrometry (HRMS) analysis was carried out with a full scan range of 50-1200 at a low collision energy of 3 V and 0.2 second scan time to determine the molecular ions of the drug related peaks. A full scan at high collision energy of 15 to 50 V and 0.2 second scan time was also run at the same time to generate the product ion spectra of the ions of interest for structural elucidation. The internal reference solution consisting of 1 μg/mL Leucine Enkephalin peptide in HPLC grade water was continually infused (10 μL/min) via the lock spray interface. This afforded ions of 278.1141 and 556.2771 and were selected for the lockmass. The source and desolvation temperatures were 110°C and 550°C, respectively, while the cone and desolvation gas flow (L/h) were 20 and 500, respectively. The capillary voltage applied was 3.0 kV and the sampling cone voltage was 39 V.
